# The Role of Parental Monitoring in the Relationships Among Academic Motivation, School Engagement, and Dropout Intention: A Two-Wave Study of Italian Adolescents

**DOI:** 10.3390/bs16050687

**Published:** 2026-04-30

**Authors:** Gaetana Affuso, Nicola Picone, Ugo Pace, Maddalena Pannone, Anna Zannone, Azzurra Giuseppa Maria Alù, Alda Troncone, Gaia Caldarelli, Stefania Cella, Daniele Abronzino, Arianna Vozza, Simona Angelini, Andrea De Matteis, Sara Murgia, Miriana Vicale, Alessia Passanisi, Lucia Di Martino, Dario Bacchini

**Affiliations:** 1 Department of Psychology, University of Campania “Luigi Vanvitelli”, 81100 Caserta, Italy; nicola.picone@unicampania.it (N.P.); magdipan@hotmail.it (M.P.); alda.troncone@unicampania.it (A.T.); gaia.caldarelli@unicampania.it (G.C.); stefania.cella@unicampania.it (S.C.); daniele.abronzino@studenti.unicampania.it (D.A.); arianna.vozza@studenti.unicampania.it (A.V.); simona.angelini@studenti.unicampania.it (S.A.); andrea.dematteis1@studenti.unicampania.it (A.D.M.); sara.murgia@studenti.unicampania.it (S.M.); miriana.vicale@studenti.unicampania.it (M.V.); 2 Human and Social Sciences, University Kore of Enna, 94100 Enna, Italy; ugopax@gmail.com (U.P.); azzurragiuseppamaria.alu@unikorestudent.it (A.G.M.A.); alessia.passanisi@unikore.it (A.P.); 3 Department of Humanistic Studies, University of Naples “Federico II”, 80133 Napoli, Italy; zannoneanna@gmail.com (A.Z.); dimartinolucia00@gmail.com (L.D.M.); dario.bacchini@unina.it (D.B.)

**Keywords:** school dropout, adolescents, parental monitoring, academic motivation, school engagement

## Abstract

School dropout is an issue that requires the attention of institutions. Related research indicates that both family (e.g., parental monitoring) and personal (e.g., academic motivation and school engagement) factors affect adolescents’ decision to quit school. As no studies have jointly examined these variables in Italian adolescents over time, this two-wave study aimed to investigate the role of parental monitoring in the relationships among academic motivation, school engagement, and dropout intention. This study enrolled 377 adolescents (boys = 178; *M*_age_ = 14.41, *SD*_age_ = 0.72) from two public upper secondary schools in Italy, and followed them over 6 months from November 2024 (T0) to May 2025 (T1). They completed a questionnaire at T0 and T1 comprising the following measures: parental monitoring, academic motivation, school engagement, and dropout intention. Structural equation modelling showed a good fit to the data, *χ*^2^_(54)_ = 84.589, *p* = 0.005, RMSEA = 0.04 [0.02–0.05], CFI = 0.99, TLI = 0.98, SRMR = 0.02. At T0, parental monitoring was positively associated with academic motivation and school engagement and negatively associated with dropout intention. A positive reciprocal association was observed between academic motivation and school engagement at T0 and T1. Academic motivation and school engagement at T0 were negatively associated with dropout intention at T1. Parental monitoring at T0 had a significant indirect effect on dropout intention at T1 via academic motivation and school engagement at T0. These findings suggest that interventions targeting family and personal factors may reduce school dropout rates among adolescents.

## 1. Introduction

As highlighted by the United Nations Educational, Scientific and Cultural Organization (UNESCO, 2026), many children and youths worldwide are out of school, with secondary education most affected. This issue requires policymakers’ attention, as school dropout entails personal and social consequences (Brunello & De Paola, 2014). Youths who have dropped out of school report high levels of poor physical and mental health, unemployment, reliance on government aid, and problems with the law later in life (Lansford et al., 2016; Ramsdal et al., 2018). Although previous studies have identified risk and protective factors for school dropout (e.g., low socioeconomic status or teacher support; Lansford et al., 2016; Tvedt et al., 2021), further research is needed to reveal other social and personal factors involved in school dropout.

In this regard, family context (e.g., parental monitoring) and personal factors (e.g., academic motivation and school engagement) may be central to adolescents’ intention to quit school. Earlier studies have shown that a lack of parental involvement in children’s education can lead to school dropout (Marlow & Rehman, 2021; Paul et al., 2021). Research also indicates that intrinsically motivated students tend to perform better academically and are less likely to drop out (Jeno et al., 2018; Messerer et al., 2023). In addition, low levels of school engagement have been linked to school dropout (Gubbels et al., 2019). Moreover, previous longitudinal studies have shown that parental support influences students’ academic motivation, which, in turn, affects their dropout intention (Alivernini & Lucidi, 2011), and that the relationship between parental monitoring and school dropout intention is mediated by students’ engagement (Blöndal & Adalbjarnardóttir, 2014). Lastly, reciprocal relationships have been demonstrated among satisfaction of basic psychological needs at school, school engagement, and academic achievement in primary school students (Y. Wang et al., 2019).

Therefore, this study aims to examine the role of parental monitoring in the relationships among academic motivation, school engagement, and school dropout intention within a sample of Italian adolescents over 6 months. It also investigates the roles of parental education, gender, age, and prior academic achievement in these relationships. Compared to the previous studies, and to our knowledge, this study is the first to combine the aforementioned variables; examine their temporal associations among Italian adolescents attending public upper secondary schools; and explore the reciprocal influences of academic motivation, school engagement, and dropout intention. Since secondary education is characterised by high dropout rates (UNESCO, 2026), the findings can be useful for designing interventions to reduce school dropout among adolescents, in line with Goal 4 of the UNESCO (2017) 2030 Agenda, which addresses inclusive and equitable quality education and lifelong learning opportunities for all.

### 1.1. Ecological Systems and School Outcomes

Ecological systems theory emphasises that human development is shaped by several systems and their interactions (Bronfenbrenner, 1977, 1994). This framework posits a layer of systems, ranging from distal to proximal in relation to the individual. Specifically, individuals are embedded in and influenced by the macrosystem (e.g., social and legal factors), the exosystem (e.g., mass media), the mesosystem (e.g., relationships between parents and teachers), and the microsystem (e.g., school and family), as well as the chronosystem, which represents the historic and temporal dimension that passes through all systems. Therefore, students’ academic trajectories may be affected not only by settings that directly surround them, such as family, but also by more distal settings, including the availability of educational resources in their communities (Ipinge & Seroto, 2024).

Regarding the school environment, Astor and Benbenishty (2019) further developed an ecological framework that emphasises the school’s role. They postulated that both external and internal school factors, along with their reciprocal influences, should be considered when examining adolescents’ academic outcomes. They noted that external school factors, including sociodemographic characteristics (e.g., age and gender), mass media, communities, and families, as well as internal school factors, including school social and organisational climate, may impact students’ lives. Specifically, internal factors may act as mediators or moderators of external factors, thereby changing them (e.g., a successful school may positively impact its local community). Although Astor and Benbenishty focused their discussion on school violence, bullying, and safety, this framework can also be applied to other outcomes. For instance, previous research has shown that parental support (external) and positive school climate (internal) contribute to students’ academic motivation and achievement (Daily et al., 2019; Guo et al., 2025; Song et al., 2015; Verner-Filion et al., 2023).

In the context of this study, these ecological frameworks provide a useful lens for examining the effects of family (parental monitoring and education) and personal (age, gender, prior academic achievement, academic motivation, and school engagement) factors on adolescents’ school dropout intention.

### 1.2. The Role of Family Context in School Dropout

Regarding the role of family context, Huisman and Smits (2015) found that, across 30 countries, children’s school dropout rates were primarily explained by family variables, including parents’ education, occupational status, and income. Similarly, Patel et al. (2018) found that girls living in poor areas and with uneducated mothers were at high risk of school dropout. Furthermore, as highlighted by an Italian report on school dropout (Autorità Garante per L’infanzia e L’adolescenza, 2022), students with foreign citizenship and those from families with unemployed or less-educated parents (who hold only a lower secondary school diploma) are less likely to remain in school. Overall, the family’s socioeconomic status, often measured through parental education and income, is a risk factor for school dropout (Lansford et al., 2016; Winding & Andersen, 2015).

Within the family context, it is crucial to account for specific practices such as parental monitoring, defined as the extent to which parents are aware of their children’s activities and whereabouts, and provide them with a structured environment (Dishion & McMahon, 1998). Notably, parental monitoring is a protective factor against a wide range of outcomes among adolescents, including depression, anxiety, substance use, and antisocial behaviours (Bacchini et al., 2011; Li et al., 2000). Parents’ involvement positively affects their children’s development when it enhances their autonomy (Rodríguez-Meirinhos et al., 2020). Indeed, self-determination theory (SDT; Ryan & Deci, 2000) can explain the role of parental monitoring in the adjustment of children and adolescents. It postulates that individuals’ well-being depends on the satisfaction of three basic psychological needs: competence, autonomy, and relatedness. Regarding parental control, Cao et al. (2022) reported that behavioural control rather than psychological control was associated with satisfying these needs, which, in turn, fostered positive development.

Regarding academic outcomes, parental monitoring serves as a dual-action mechanism: it simultaneously mitigates antisocial conduct and promotes prosocial behaviour and academic achievement (Top et al., 2017). Its critical nature is further evidenced by its role as a primary determinant of school retention. For example, significant deficits in parental monitoring have been directly linked to increased risk of school dropout, even among high-risk populations such as juvenile offenders (Fernández-Suárez et al., 2016). Longitudinal research also seems to confirm these findings. For instance, Blöndal and Adalbjarnardóttir (2014) investigated the role of parental behaviours in school dropout in a sample of adolescents followed over 8 years, finding that reduced parental supervision at age 14 years was associated with a greater risk of school dropout by age 22 years. Lastly, a meta-analysis highlighted an association between negative parental behaviours, including low monitoring, and primary and secondary school dropout (Marlow & Rehman, 2021).

### 1.3. The Role of Personal Factors in School Dropout

Regarding the role of sociodemographic factors, a meta-analysis found that girls typically perform better than boys across different school subjects (Voyer & Voyer, 2014). Some scholars have explained this finding by attributing girls’ academic success to factors such as greater motivation and attitudes toward school (Van Houtte, 2004; Vecchione et al., 2014). As prior academic achievement predicts the likelihood of school dropout (Balkis, 2018), this gender difference may account for the higher school dropout rates observed among boys (UNESCO, 2022). In addition to gender, the potential impact of age should be considered, as secondary education is particularly affected by dropout rates (UNESCO, 2026). Students are more likely to drop out as they progress through the school system (Eurostat, 2025), in part due to the limits of compulsory schooling (Wenger, 2002).

Among school-related factors, academic motivation and school engagement are often investigated in research on school dropout (Anttila et al., 2023; M. T. Wang & Fredricks, 2014). Motivation is conceptualised into several dimensions based on its source: intrinsic motivation refers to the drive deriving from one’s own pleasure in doing something, whereas extrinsic motivation refers to the drive deriving from external sources of reinforcement (Deci & Ryan, 1985). Students may be motivated by their interest and pleasure in studying or by external factors, such as school grades or career opportunities. Within the SDT framework (Deci & Ryan, 1985; Ryan & Deci, 2000), Vallerand (1997) further developed a hierarchical model arguing that several types of motivation can emerge along a continuum from intrinsic motivation (the most self-determined or autonomous form) to amotivation (the least self-determined or autonomous form), with more self-determined forms leading to positive cognitive, affective, and behavioural outcomes.

Regarding academic factors, cross-sectional (Manganelli et al., 2019) and longitudinal (Affuso et al., 2023; Niehaus et al., 2012; Verner-Filion et al., 2023) studies have found a positive association between academic motivation and achievement among secondary school and university students. With specific reference to school dropout, one longitudinal study showed that self-determined motivation had a stronger impact on adolescents’ school dropout intention than other factors, such as social support (e.g., from parents and teachers), academic achievement, and self-efficacy (Alivernini & Lucidi, 2011). Similarly, Rump et al. (2017) found that students’ self-determined forms of motivation (intrinsic and identified regulation) were strongly associated with school dropout intention.

Recently, scholars have shown increasing interest in school engagement. Drawing on positive psychology (Seligman & Csikszentmihalyi, 2000), Schaufeli et al. (2002) defined engagement as ‘a positive, fulfilling, and work-related state of mind that is characterised by vigor, dedication, and absorption’ (p. 74). The relevance of this psychological variable extends beyond the educational context, as this construct has proven crucial in both organisational (Shimazu et al., 2010) and academic (Dimitriadou et al., 2020) settings. Cognitive and emotional engagement have also been associated with academic performance among university students (Gutiérrez & Tomás, 2019). Furthermore, longitudinal evidence suggests that engagement and prior academic achievement are significant negative predictors of school dropout (Fall & Roberts, 2012). Similarly, a literature review revealed that low school engagement is a risk factor for school absenteeism and dropout (Gubbels et al., 2019). Lastly, it is notable that motivation and engagement may influence one another, as a previous study found that motivational beliefs were associated with adolescents’ cognitive, emotional, and behavioural engagement (M.-T. Wang & Eccles, 2013).

### 1.4. The Role of Parental Monitoring in the Relationships Among Academic Motivation, School Engagement, and School Dropout Intention

Empirical evidence indicates that parental monitoring’s influence on academic success is often indirect, operating through the development of internal resources such as self-motivation and self-efficacy (Affuso et al., 2017, 2023). This mediational pathway is further supported by Cheung and Pomerantz (2012), who showed that parental involvement was related to better school grades among children through its effects on motivation and self-efficacy over time. A similar pattern emerges regarding school engagement. Longitudinal research has identified school engagement as a crucial bridge between early parental supervision and long-term educational outcomes. For instance, the protective effect of monitoring at age 14 years against subsequent dropout is significantly mediated by the student’s level of engagement a year later (Blöndal & Adalbjarnardóttir, 2014). In this study’s context, this finding is particularly relevant because Italian adolescents typically enter upper secondary school at age 14 years, a transitional period during which the risk of dropout increases (Perico E Santos, 2023). Even in non-mediational frameworks, a robust association persists between parental monitoring and heightened school engagement, although this relationship may be nuanced by specific family characteristics (Malczyk & Lawson, 2017).

Despite the scarcity of studies testing mediational models, the existing literature indicates that parental monitoring may affect adolescents’ school dropout intention over time, mediated by academic motivation and school engagement.

### 1.5. The Italian Context: School System and Dropout

The Italian school system comprises 13 grades divided into primary (1st–5th), lower secondary (6th–8th), and upper secondary education (9th–13th). Students enter upper secondary education (high school) in 9th grade, when they are usually aged 14 years old, and complete it by passing a final state exam in 13th grade, when they are usually 18 years old. Although upper secondary education lasts 5 years, schooling is compulsory only from ages 6 to 16 years, when students are in the 10th grade.

Although school dropout rates in Italy have decreased over the past few years, they remain higher than those of other European countries and represent a significant problem (Bonaiuti et al., 2025; Istituto Nazionale di Statistica [ISTAT], 2025a). In 2024, the proportion of individuals aged 25–64 years with at least a high school diploma was 66.7% in Italy, below the European average of 80.5%, and was even lower in southern regions (Istituto Nazionale di Statistica [ISTAT], 2025a).

In upper secondary education, the first two years (9th and 10th grades) constitute a high-risk period for dropout (Ministero dell’Istruzione e del Merito, 2023). Specifically, the early school leaving rate was 9.8% in 2024. In addition, gender and geographic differences have been observed, as boys and students from Southern regions are at greater risk of dropping out of high school (12.2.% and 12.4%, respectively). These data mirror those from the city of Naples (Campania region, Southern Italy), where 10.25% of upper secondary students dropped out of school or were at risk of school dropout (due to an elevated number of absences) between 2023 and 2024 (Direzione Generale dell’Ufficio Scolastico Regionale per la Campania, 2024). Similarly, the Campania region was found to be among the Italian regions with the highest proportion of young people who are neither in employment nor in education and training (Vegliante et al., 2024). Other differences across school types have also been identified, with students attending private schools at particular risk of school dropout (Ministero dell’Istruzione e del Merito, 2023). Furthermore, regarding socioeconomic conditions, Istituto Nazionale di Statistica [ISTAT] (2025b) reported that 43.6% of children in Southern Italy experience poverty and social exclusion (e.g., living in a family that cannot afford an internet connection or going on holiday). This observation is particularly critical, as low socioeconomic status is a predictor of school dropout (Lansford et al., 2016), and participants in this study were drawn from two public upper secondary schools in Naples, Italy.

### 1.6. The Present Study

Given the aforementioned considerations, the present study aims to examine the role of parental monitoring in the relationships among academic motivation, school engagement, and school dropout intention in a sample of Italian adolescents followed over 6 months from November 2024 (T0) to May 2025 (T1). It also investigates the roles of parental education, gender, age, and prior academic achievement in these relationships.

Specifically, it hypothesises the following:

1. Parental monitoring at T0, parental education, gender (male = 1 and female = 2), and prior academic achievement will be positively associated with academic motivation and school engagement and negatively associated with school dropout intention at T0 and T1, whereas age will be negatively associated with the former and positively associated with the latter.
2. Over the two time points (T0 and T1), academic motivation and school engagement will be reciprocally and positively associated, whereas school dropout intention will be reciprocally and negatively associated with both.
3. Parental monitoring at T0 will have an indirect negative effect on school dropout intention at T1, mediated by academic motivation and school engagement at T0.

## 2. Materials and Methods

### 2.1. Participants

The study participants were part of a larger Italian research project, ‘PRO-SELF’, which aims to reduce risk-taking behaviours among adolescents by promoting self-regulation. In this study, participants were drawn from two public upper secondary schools with different specialisations in the city of Naples in Southern Italy, an area where youths are more likely to live in socioeconomically disadvantaged families (Istituto Nazionale di Statistica [ISTAT], 2025b). The study sample comprised 377 adolescents, 199 females (52.8%) and 178 males (42.7%), with a mean age of 14.41 years (*SD* = 0.72), enrolled in the first and second year of upper secondary school (9th and 10th grades, respectively). The participants were asked to complete a questionnaire twice over 6 months, in November 2024 (T0) and May 2025 (T1). Regarding their parents, 51.6% of mothers and 57.9% of fathers had a low level of education, 40.3% of mothers and 36.1% of fathers had a middle level of education, and 8.1% of mothers and 6.0% of fathers had a high level of education.

### 2.2. Procedure

Before starting data collection, we obtained written consent from the school principals, participating adolescents, and their parents. The participants completed the questionnaire during school hours under the supervision of the researchers. This study was approved by the Research Ethics Committee of Psychological Research of the Department of Humanities Studies, University of Naples “Federico II”, and was conducted in accordance with the American Psychological Association’s ethical standards for research involving human subjects.

### 2.3. Measures

#### 2.3.1. Sociodemographic Factors

The participants self-reported their age, gender (1 = male, 2 = female), and their parents’ education levels (ranging from 1 [primary school] to 5 [university degree]). In the analyses, the latent variable for parental education was composed of the parents’ education levels.

#### 2.3.2. Prior Academic Achievement

The participants self-reported their final examination grade at the end of lower secondary school (8th grade). In the Italian school system, grades range from 6 (the minimum passing grade) to 10.

#### 2.3.3. Parental Monitoring

At T0, the participants completed the Parental Knowledge subscale of the Parental Monitoring Scale (Kerr et al., 2010). We used the Italian version of this scale, which has demonstrated good psychometric properties among adolescents (Miranda et al., 2012). It comprises seven items that assess how children perceive their parents’ knowledge of their activities and whereabouts (e.g., ‘How much do your parents really know who your friends are?’). Items are rated on a Likert-type scale from 1 (never) to 5 (always). In this study, the subscale had a Cronbach’s α of 0.82.

#### 2.3.4. Academic Motivation

At T0 and T1, the participants completed the Academic Motivation Scale (Vallerand et al., 1993). We used the Italian version of this scale, which has demonstrated good psychometric properties among adolescents (Alivernini & Lucidi, 2008). It comprises five subscales, each consisting of four items: Amotivation, External Motivation, Introjected Motivation, Identified Motivation, and Intrinsic Motivation. The items are potential answers to the question ‘Why do you go to high school?’, which are rated on a Likert-type scale from 0 (does not correspond at all) to 7 (corresponds exactly). The relative autonomy index (RAI; Vallerand & Ratelle, 2002) was calculated by weighting scores across subscales, with higher RAIs indicating greater self-determined motivation. In this study, Cronbach’s α for each subscale at T0 and T1 was as follows: 0.89 and 0.88 for Amotivation, 0.80 and 0.83 for External Motivation, 0.82 and 0.84 for Introjected Motivation, 0.82 and 0.85 for Identified Motivation, and 0.88 and 0.89 for Intrinsic Motivation.

#### 2.3.5. School Engagement

At T0 and T1, the participants completed the Utrecht Work Engagement Scale for Students (Carmona-Halty et al., 2019). We used the Italian version of this scale, which has demonstrated good psychometric properties among adolescents (Fiorilli et al., 2014). It comprises three subscales, each consisting of three items: Vigour, Dedication, and Absorption. Items (e.g., ‘When I’m doing my work as a student, I feel bursting with energy’) are rated on a Likert-type scale from 0 (never) to 6 (always), with higher scores indicating greater school engagement. In this study, Cronbach’s α for each subscale at T0 and T1 was as follows: 0.87 and 0.81 for Vigour, 0.88 and 0.91 for Dedication, and 0.75 and 0.72 for Absorption. Because the three subscales are highly correlated (Pearson’s *r* > 0.70), a latent variable for school engagement was used in the analyses.

#### 2.3.6. School Dropout Intention

At T0 and T1, the participants completed three items assessing their school dropout intention (e.g., ‘I sometimes consider dropping out of school’; ‘I intend to drop out of school’; ‘I sometimes feel unsure about continuing my studies year after year’; Hardre & Reeve, 2003). We used the Italian version of this scale, which has demonstrated good psychometric properties among adolescents (Alivernini & Lucidi, 2011). They were asked to rate their agreement with each item on a Likert-type scale from 1 (not at all) to 7 (very much so). Higher scores indicate greater school dropout intention. In this study, Cronbach’s α was 0.83 at T0 and 0.86 at T1.

### 2.4. Data Analysis

All the analyses were conducted in SPSS (version 21; IBM Corp., Armonk, NY, USA) and Mplus (version 7.4) and *p*-values < 0.05 were considered statistically significant. All variables measured at T0 were compared between the participants who dropped out and those who did not using multivariate analysis of variance (MANOVA). Associations between variables were assessed pairwise using Pearson’s correlation coefficient (*r*). To explore interactions among the study variables over time, a structural equation model (SEM) was fitted, with parental monitoring at T0, parental education, age, gender, and prior academic achievement included as independent variables, and academic motivation, school engagement, and school dropout intention included as dependent variables. Maximum likelihood estimates were used (Muthén & Muthén, 2017) and the indirect effects of independent variables at T0 on dependent variables at T1 were computed using the indirect effect test implemented in Mplus (version 7.4). How well the model fitted the data was assessed using the following indices: the comparative fit index (CFI; Bentler, 1990), the Tucker–Lewis index (TLI; Tucker & Lewis, 1973), the root-mean-square error of approximation (RMSEA; Brown & Cudeck, 1993), and the standardised root-mean-square residual (SRMR; Hu & Bentler, 1999). Acceptable fit to the data is indicated by CFI and TLI ≥ 0.90 and RMSEA and SRMR ≤ 0.08 (Hu & Bentler, 1999).

## 3. Results

### 3.1. Attrition Analysis and Descriptive Statistics

In the attrition analysis, 11/377 (2.92%) participants dropped out during the study period: 9 males (2.38%) and 2 females (0.53%). Little’s missing completely at random test (Little & Rubin, 2002) was significant (*χ*^2^ = 452.477, *df* = 404, *p* = 0.048), indicating that the missing data were not entirely random. Therefore, full information maximum likelihood estimation was used to handle missing data in SEM. Nonetheless, the mean scores for all variables measured at T0 did not differ significantly between participants who dropped out and those who did not, Wilks’s *λ* = 0.99, *F*_(6)_ = 0.39, *p* = 0.880.

The correlations, means, and standard deviations are reported in Table 1. Parental monitoring, academic motivation, school engagement (vigour, dedication, and absorption), and prior academic achievement significantly and positively correlated with each other across T0 and T1. School dropout intention significantly and negatively correlated with parental monitoring, academic motivation, school engagement (vigour, dedication, and absorption), and prior academic achievement across T0 and T1. Gender significantly and negatively correlated with father’s education, and significantly and positively correlated with academic motivation at T0 and T1, and school engagement (absorption) at T0. Age significantly and negatively correlated with parental monitoring at T0 and school dropout intention at T1. Father’s education significantly and positively correlated with mother’s education, prior academic achievement, and school engagement (vigour and dedication) at T0, and significantly and negatively correlated with school dropout intention at T0 and T1. Lastly, mother’s education significantly and positively correlated with prior academic achievement, academic motivation at T0 and school engagement (dedication) at T1, and significantly and negatively correlated with school dropout intention at T0 and T1.

### 3.2. Direct and Indirect Associations Between Variables

The SEM exhibited a good fit to the data, *χ*^2^_(54)_ = 84.589, *p* = 0.005, RMSEA = 0.04 [0.02–0.05], CFI = 0.99, TLI = 0.98, and SRMR = 0.02. Overall, the SEM explained 58% of the variance in academic motivation at T1, 65% of the variance in school engagement at T1, and 51% of the variance in school dropout intention at T1.

**Figure 1:**
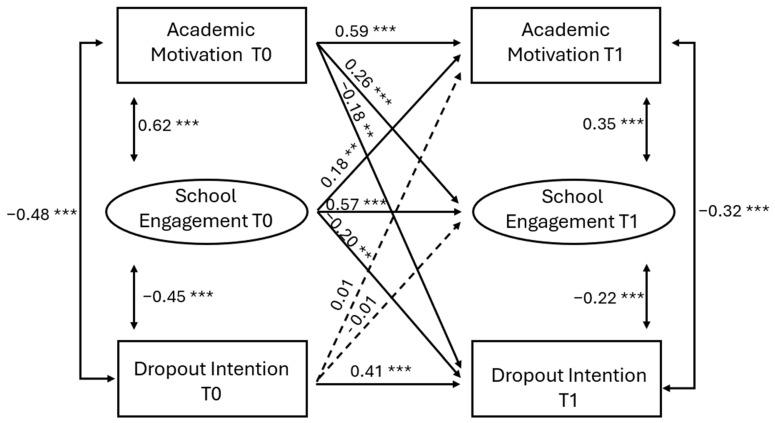
Reciprocal relationships between academic motivation, school engagement, and dropout intention across time. Note: Standardised path coefficients. ** *p* < 0.01, *** *p* < 0.001. To simplify, the paths from gender, age, prior academic achievement, parental education, and parental monitoring to all other variables were omitted and reported in Table 2.

The associations between the study variables are reported in Table 2 and illustrated in Figure 1. Regarding sociodemographic factors, gender was significantly and positively associated with academic motivation at T0, while age was significantly and negatively associated with school dropout intention at T1. Prior academic achievement was significantly and positively associated with school engagement at T0 and T1, and academic motivation at T1. Parental education was not significantly associated with any variable. Parental monitoring at T0 was significantly and positively associated with academic motivation and school engagement at T0, and significantly and negatively associated with school dropout intention at T0. Regarding the reciprocal associations, only academic motivation and school engagement were significantly and positively associated across T0 and T1. Academic motivation and school engagement at T0 were significantly and negatively associated with school dropout intention at T1. Lastly, all the autoregressive effects were significant across T0 and T1.

The indirect effects are reported in Table 3. Parental monitoring at T0 had a significant and negative indirect effect on school dropout intention at T1, mediated by academic motivation, school engagement, and school dropout intention at T0, as well as a significant and positive indirect effect on academic motivation and school engagement at T1, mediated by academic motivation and school engagement at T0. As gender and prior academic achievement were significantly associated with academic motivation and school engagement at T0, respectively, their indirect effects were also evaluated. Gender had a significant and positive indirect effect on academic motivation and school engagement at T1, and a significant and negative indirect effect on school dropout intention at T1, mediated by academic motivation at T0. Prior academic achievement had a significant and positive indirect effect on school engagement at T1, mediated by school engagement at T0.

## 4. Discussion

This study investigated the roles of family context and personal factors in school dropout intention among Italian adolescents followed over 6 months. Specifically, it examined the role of parental monitoring in the relationships among academic motivation, school engagement, and school dropout intention, as well as those of parental education, gender, age, and prior academic achievement.

Consistent with our hypothesis, parental monitoring at T0 was positively associated with academic motivation and school engagement and negatively associated with school dropout intention at T0. In addition, parental monitoring had a negative indirect effect on school dropout intention at T1, mediated by academic motivation, school engagement, and school dropout intention at T0. Moreover, parental monitoring had a positive indirect effect on academic motivation and school engagement at T1, mediated by academic motivation and school engagement at T0. Despite the scarcity of studies testing mediational models with these variables, our findings align with prior research (Bacchini et al., 2011; Li et al., 2000) suggesting that parental monitoring is protective against school dropout intention. They are also consistent with research showing that parental monitoring is associated with academic motivation, school engagement, and school dropout (Blöndal & Adalbjarnardóttir, 2014; Top et al., 2017). Specifically, our findings indicate that parents who are involved in their children’s lives and provide a structured environment are more likely to promote academic motivation and school engagement, which, in turn, counteract school dropout intention.

Under the SDT (Ryan & Deci, 2000), monitoring contributes to parents meeting their children’s basic psychological needs, including autonomy, competence, and relatedness (Cao et al., 2022), which may lay the foundation for the development of self-determined or autonomous forms of motivation and school engagement. Relatedly, previous studies have confirmed that basic psychological needs are positively associated with academic motivation and school engagement (Faye & Sharpe, 2008; Y. Wang et al., 2019). Notably, the measure of parental monitoring used in our study assesses parents’ behavioural practices (e.g., knowing how their children spend their money or who their children’s friends are). Thus, our findings mirror the literature, which shows that parental involvement fosters adolescents’ adjustment through behavioural rather than psychological control (Bacikova-Sleskova et al., 2024; Cao et al., 2022).

Consistent with our hypothesis and prior research (Rump et al., 2017; Zhaohui et al., 2025), academic motivation and school engagement at T0 were negatively associated with school dropout intention at T1. Thus, more self-motivated and engaged students were less likely to consider dropping out of school than less self-motivated and engaged students. Drawing on Vallerand’s (1997) model and positive psychology (Seligman & Csikszentmihalyi, 2000), our findings suggest that when students are driven by pleasure and feel energetic, enthusiastic, and absorbed in their academic activities, they are more likely to decide to remain in school. Moreover, academic motivation and school engagement reciprocally influenced each other across T0 and T1, consistent with prior research (M.-T. Wang & Eccles, 2013; Y. Wang et al., 2019) and supporting the notion that interrelations may exist between these personal factors.

Regarding the sociodemographic factors, gender was positively associated with academic motivation at T0. It also had a positive indirect effect on academic motivation and school engagement at T1 and a negative indirect effect on school dropout intention at T1, mediated by academic motivation at T0. Thus, being female was associated with greater academic motivation and school engagement, as well as lower school dropout intention. Previous studies have also reported gender differences, with girls reported to have an academic advantage over boys (e.g., better school grades; Voyer & Voyer, 2014), which would account for the higher school dropout rates among boys (Borgna & Struffolino, 2017). Indeed, although historically, women had to struggle to achieve academic success for social reasons (e.g., patriarchy), there is currently a large number of boys who are out of school (UNESCO, 2022). This finding may be explained by several factors, including greater academic motivation among girls (Bugler et al., 2015), consistent with our findings.

As our study sample comprised two age groups (students enrolled in 9th and 10th grades), age could be examined as a variable. Contrary to our hypothesis, we found that age was negatively associated with school dropout intention at T1. Thus, older adolescents were less likely to consider dropping out of school, contradicting data indicating that students are more likely to drop out as they progress through school grades (Eurostat, 2025). Stearns and Glennie (2006) observed that economic pressure (e.g., job-seeking) may influence school dropout intention among older students. Therefore, further research is needed to clarify the relationship between age and the intention to drop out of school.

Lastly, the final examination grade at the end of lower secondary school (8th grade) was positively associated with school engagement at T0 and T1, and academic motivation at T1. It also had a positive indirect effect on school engagement at T1, mediated by school engagement at T0. This finding aligns with the literature on the predictive value of prior academic achievement (Balkis, 2018; Tvedt & Bru, 2023), indicating that students who perform worse on school subjects may be at greater risk of school dropout and should be targets for preventive interventions.

Overall, our findings showed that parental monitoring, along with academic motivation and school engagement, is protective against school dropout. They also showed that other personal factors, such as gender, age, and prior academic achievement, affect school dropout. Thus, in light of ecological frameworks (Astor & Benbenishty, 2019; Bronfenbrenner, 1977, 1994), our findings emphasise the relationships among family system and personal factors and their influence on adolescents’ academic adjustment.

### 4.1. Study Limitations

Our study had limitations that should be acknowledged. Firstly, although it followed participants over 6 months, the inclusion of additional measurement points might have strengthened the findings. For instance, they would enable a clearer understanding of the relationships among variables through a within-person analytical approach across multiple time points. Secondly, it examined single-informant data (e.g., student-reported measures), whereas multi-informant data could have provided additional information about the relationships among the study variables (e.g., parent-reported measures). For instance, including diverse sources of information would have allowed us to examine whether consistency between them emerged, as well as their respective effects on school dropout intention. Thirdly, although both family and personal factors were examined, including additional variables (e.g., teacher support) would have allowed us to determine whether certain factors exert a stronger effect on school dropout intention. Fourthly, the study sample comprised adolescents residing in a Southern Italian region characterised by high school dropout rates and socioeconomically disadvantaged conditions (Istituto Nazionale di Statistica [ISTAT], 2025a, 2025b), which may limit the generalisability of our findings to the broader Italian population. Nonetheless, school dropout is a widespread issue in Italy and other cultural contexts (Bonaiuti et al., 2025; UNESCO, 2026). Therefore, further longitudinal research is needed to overcome these limitations.

### 4.2. Practice and Policy Recommendations

Nevertheless, this study also had notable strengths. Firstly, to our knowledge, this study is the first to examine the role of parental monitoring in the relationships among academic motivation, school engagement, and school dropout intention among Italian adolescents over 6 months. Moreover, it is the first to examine the reciprocal influences among academic motivation, school engagement, and school dropout intention over time among adolescents. Secondly, it examined the roles of parental education, gender, age, and prior academic achievement in these relationships.

Finally, our findings have practical implications in line with Goal 4 of the UNESCO (2017) 2030 Agenda, as high dropout rates have been found in secondary education worldwide (UNESCO, 2026) and in the context of Southern Italy, where this study was conducted (Direzione Generale dell’Ufficio Scolastico Regionale per la Campania, 2024). Specifically, our study highlights the need for interventions on family and personal factors. For instance, enhancing parents’ involvement in their children’s lives may increase their academic motivation and school engagement, ultimately leading to better academic outcomes and reducing dropout rates during secondary school. Indeed, school psychologists and educators may involve parents in educational interventions to provide them with strategies to enhance the monitoring and supervision of their children. They may also help parents reflect on the contexts in which monitoring should be demonstrated (e.g., knowing about their children’s behaviour at school or how their children spend money) and on the possibility of involving other adults when they are unavailable to monitor their children (Schwarz-Torres et al., 2025). Moreover, increasing students’ academic motivation may promote their school engagement and vice versa. Indeed, other potential interventions may include cooperative learning, which has been shown to positively affect students’ self-determined motivation and engagement (Fernandez-Rio et al., 2017; Van Ryzin & Roseth, 2021). Overall, interventions should be conducted with the understanding that low-achieving adolescents and boys may be at greater risk of school dropout than others. Efforts towards these prevention actions should be a key priority for policymakers, given the high school dropout rates (UNESCO, 2026) and the associated personal and social costs (Brunello & De Paola, 2014).

## Figures and Tables

**Table 1:** Means, SDs, and correlations of the study variables.

	1	2	3	4	5	6	7	8	9	10	11	12	13	14	15	16
1. Gender	1															
2. Age	−0.04	1														
3. Prior Academic Achievement	0.06	0.05	1													
4. Father’s education	−0.12 *	−0.07	0.26 ***	1												
5. Mother’s education	−0.04	−0.05	0.15 *	0.47 ***	1											
6. Parental monitoring T0	0.10	−0.12 *	0.26 ***	0.11	0.06	1										
7. Motivation T0	0.23 ***	0.06	0.23 ***	0.07	0.14 *	0.46 ***	1									
8. Engagement-VI T0	0.09	0.03	0.19 **	0.18 **	0.06	0.40 ***	0.61 ***	1								
9. Engagement-DE T0	0.06	−0.01	0.27 ***	0.16 **	0.09	0.40 ***	0.65 ***	0.72 ***	1							
10. Engagement-AB T0	0.13 *	−0.01	0.22 ***	0.06	0.01	0.40 ***	0.60 ***	0.74 ***	0.74 ***	1						
11. Dropout Intention T0	−0.01	−0.08	−0.18 **	−0.15 **	−0.16 **	−0.39 ***	−0.57 ***	−0.47 ***	−0.49 ***	−0.42 ***	1					
12. Motivation T1	0.19 **	0.07	0.28 ***	0.06	0.04	0.37 ***	0.75 ***	0.52 ***	0.57 ***	0.53 ***	−0.44 ***	1				
13. Engagement-VI T1	0.09	0.08	0.22 ***	0.10	0.05	0.37 ***	0.55 ***	0.69 ***	0.57 ***	0.61 ***	−0.42 ***	0.56 ***	1			
14. Engagement-DE T1	0.06	0.04	0.30 ***	0.11	0.11 *	0.34 ***	0.65 ***	0.56 ***	0.71 ***	0.56 ***	−0.44 ***	0.67 ***	0.74 ***	1		
15. Engagement-AB T1	0.04	0.05	0.25 ***	0.07	0.05	0.35 ***	0.55 ***	0.55 ***	0.55 ***	0.61 ***	−0.39 ***	0.58 ***	0.73 ***	0.74 ***	1	
16. Dropout Intention T1	−0.05	−0.12 *	−0.26 ***	−0.14 *	−0.20 ***	−0.36 ***	−0.59 ***	−0.4 ***	−0.53 ***	−0.47 ***	0.66 ***	−0.62 ***	−0.51 ***	−0.57 ***	−0.46 ***	1
M	1.56	14.40	6.24	2.40	2.52	3.74	17.43	2.20	2.95	2.42	5.48	15.64	2.23	2.88	2.34	5.58
SD	0.50	0.70	1.32	0.74	0.70	0.82	21.30	1.43	1.50	1.47	2.84	21.47	1.36	1.59	1.38	2.92

Note: Gender: 1 male, 2 females. Engagement-VI = Engagement—Vigour. Engagement-DE = Engagement—Dedication. Engagement-AB = Engagement—Absorption. Listwise participants. * *p* < 0.05, ** *p* < 0.01, *** *p* < 0.001.

**Table 2:** Standardised effects of gender, age, prior academic achievement, parental education, and parental monitoring on motivation, engagement, and dropout intention at T0 and T1.

	Motivation T0	Engagement T0	Dropout Intention T0	Motivation T1	Engagement T1	Dropout Intention T1
Gender	0.19 ***	0.06	0.04	0.04	−0.01	0.01
Age	0.07	0.06	−0.08	0.01	0.02	−0.09 *
Prior academic achievement	0.09	0.13 *	−0.07	0.13 *	0.09 *	−0.08
Parental education	0.05	0.05	−0.11	−0.03	−0.06	−0.03
Parental monitoring T0	0.42 ***	0.44 ***	−0.36 ***	−0.01	0.01	0.01

Note: Gender: 1 male, 2 females. * *p* < 0.05, *** *p* < 0.001.

**Table 3:** Standardised indirect effects.

Independent Variables	Mediation Variables	Dependent Variables
		Motivation T1
Parental monitoring T0	via motivation at T0	0.24 ***
	via engagement at T0	0.08 **
		Engagement T1
	via motivation at T0	0.11 ***
	via engagement at T0	0.25 ***
		Dropout Intention T1
	via motivation at T0	−0.07 **
	via engagement at T0	−0.09 **
	via dropout intention T0	−0.15 **
		Motivation T1
Prior academic achievement	via engagement at T0	0.02
		Engagement T1
	via engagement at T0	0.07 *
		Dropout Intention T1
	via engagement at T0	−0.03
		Motivation T1
Gender	via motivation at T0	0.11 ***
		Engagement T1
	via motivation at T0	0.05 **
		Dropout Intention T1
	via motivation at T0	−0.03 *

Note: * *p* < 0.05, ** *p* < 0.01, *** *p* < 0.001.

## Data Availability

Data are available from the corresponding author on reasonable request.
